# Supplementary material for: Machine learning assisted interferometric structured illumination microscopy for dynamic biological imaging
Source: Nat Commun. 2022 Dec 21;13:7836. doi: 10.1038/s41467-022-35307-0 (PMC9772218; doi:10.1038/s41467-022-35307-0)
Supplement: Supplementary file 1 — Supplementary information [file 41467_2022_35307_MOESM1_ESM.pdf]

Machine learning assisted interferometric structured illumination microscopy for dynamic biological imaging – Supplementary information

Edward N. Ward, Lisa Hecker, Charles N. Christensen, Jacob R. Lamb, Meng Lu, Luca Mascheroni, Chyi Wei Chung, Anna Wang, Christopher J. Rowlands, Gabriele S. Kaminski Schierle, Clemens F. Kaminski

**Supplementary Figure 1**	**Optical path for fringe generation**
**Supplementary Note 1**	**Hardware Description**
**Supplementary Figure 2**	**Influence of path length on modulation depth**
**Supplementary Figure 3**	**Optimization of pattern orientation**
**Supplementary Figure 4**	**Pattern phase optimization**
**Supplementary Figure 5**	**Effects of phase shifting on reconstruction quality**
**Supplementary Figure 6**	**Resolution estimation on single beads**
**Supplementary Figure 7**	**Resolution estimation with Fourier ring correlation**
**Supplementary Figure 8**	**Schematic of data generation and network training for ML-SIM.**
**Supplementary Figure 9**	**Reconstruction performance with pattern phase error**
**Supplementary Figure 10**	**Error map comparison of reconstruction quality**
**Supplementary Figure 11**	**GUI for live ML-SIM reconstruction**
**Supplementary Figure 12**	**Proposed optical setup for 3D MAI-SIM**
**Supplementary Note 2**	**MAI-SIM alignment and calibration**
**Supplementary Note 3**	**Reconstruction methods**
**Supplementary Table 1**	**Reconstruction quality measured by RSP and RSE**

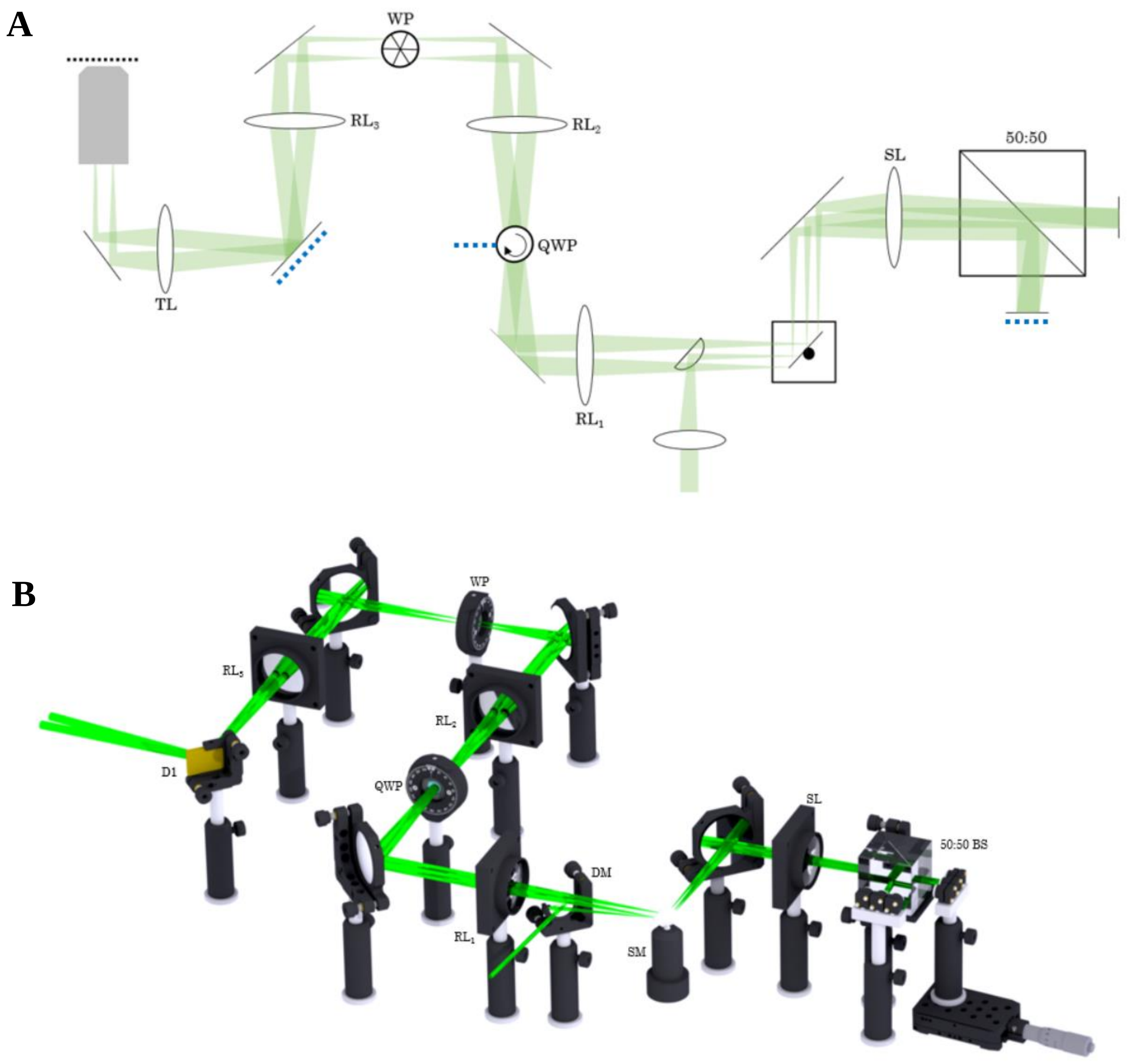

Supplementary Figure 1: Optical path for fringe generation. Simplified optical schematic (A) and rendering of typical setup (B) for MAI-SIM pattern generation. Light enters the system through a D-mirror (DM) and directed onto one of the three mirror pairs by the scan mirror (SM) and scan lens (SL). The returning beams are subsequently relayed to the back focal plane of the objective by a series of relays lenses ($RL_{1\text{-}3}$) and a tube lens (TL). A quarter waveplate (QWP) and wedged polarizer (WP) are used to optimize polarization orientation. Blue dashed lines indicate Planes conjugate to the sample plane.

Supplementary Note 1 – Hardware Description

Three laser lines are used in the MAI-SIM system with wavelengths of 491 nm (Cobolt Calypso, 200 mW), 561 nm (Oxxius SLIM-561-150, 500 mW), and 647 nm (Toptica iBeam SMART, 100 mW). These are combined coaxially using dichroic mirrors. The beam profiles are cleaned using a pinhole at the focus of a Keplerian beam expander to provide uniform illumination across the whole FOV. The expanded beam is focused onto the scan mirror (SM) (Scanlab, dynAXIS-M) using a 150 mm focal length lens (Thorlabs, AC254-150-A) and a D-mirror, (Thorlabs, PFD10-03-F01) to separate the incoming and outgoing beams of the interferometer. After reflection by the scan mirror, the beam is re-collimated by a scan lens, (Thorlabs, AC508-150-A) and subsequently enters the interferometer. A 2-inch 50:50 beam splitter cube (Thorlabs, BS031) is used for amplitude splitting and the two resulting beams each hit one of two complementary mirrors in an arrangement of 3 pairs of individually adjustable ½-inch mirrors (Thorlabs, BB05-E01). Complementary mirrors are tilted with respect to another to generate wedge fringes of the correct orientation. The small mirrors are additionally placed individually on micrometer translation stages (Thorlabs, XRN25P / XRN25C) and this enables the adjustment of the path length difference for each interferometer configuration individually to achieve optimal contrast in all orientations (Supplementary Figure 3).[1] The two beams reflected from one of the pairs pass back through the beam splitter and the scan mirror. Both beams then pass over the top of the D-mirror and are combined by a series of 150 mm focal length lenses, (Thorlabs, AC508-150-A) to form an interference pattern at an angle determined by the scan mirror position and corresponding angle of the small mirror (Main text figure 1A). The beams are then relayed through a wedged polarizer, (CODIXX, custom part). This static element ensures the polarization is correct for all orientations, while maintaining the multicolor capabilities of the system.[2] An achromatic quarter waveplate, placed in the image plane of the relay, is used to generate circularly polarized light before the beam hits the wedged polarizer. This ensures equal intensities for all fringe orientations. The beams enter the inverted microscope frame (Olympus, IX73) via a quad-band dichroic mirror, (Chroma, ZT405/488/561/640rpcv), which is from the same production batch as the dichroic mirror mounted in the microscope frame. The arrangement separates the excitation and emission light paths. Pairing these mirrors from the same batch ensures that any ellipticity introduced by birefringence of one mirror is cancelled out by the other. Thus s-polarization can be maintained for all orientations. Similar considerations apply to other conjugate mirror pairs in the optical setup.[3] The beams are focused onto the back focal plane of the objective lens (Olympus, UPLSAPO60XW) by a 300 mm focal length tube lens, (Thorlabs, AC508-300-A) and interfere in the sample plane to form a sinusoidal illumination pattern. The resulting fluorescent signal is collected by the same objective and passes through the dichroic mirror, onto an image splitting device (Cairn, Optosplit III). The image splitter consists of 2 dichroic mirrors and corresponding passband emission filters to separate individual color channels according to the

chosen fluorophores. Up to three channels are then displayed side by side on a sCMOS camera with 2048 × 2048 pixel chip and 6.5 µm × 6.5 µm pixel size (PCO edge 4.2 bi).

Synchronization in the final system is achieved by controlling the camera exposure, stage control, and mirror movement through a LabVIEW interface, while the image data was read in a separate application (Micromanager).[4] Digital I/O lines from a DAQ card (National Instruments, BNC-2110) were used to control the exposure time and monitor the data readout from the camera using the I/O lines from the camera. In parallel, the mirror position was controlled using the analogue I/O lines from the same DAQ card. We found that the time taken to move and settle the mirror at different positions is comparable to the data readout time for a 512 × 512 pixel image. The frame rate was therefore maximized by moving the mirror after the exposure had finished while the data were read from the camera. Small digital delays were added before and after mirror movement to ensure that the mirror was stationary during camera exposure. The stage was controlled using serial commands in the same LabVIEW interface. Stage movement was similarly synchronized to camera readout to maximize frame rates, although extra digital delays were needed before the following acquisition as z-movements were significantly slower than camera readout. All digital delays (see timing diagram on project page) were determined empirically by imaging a thin fluorescent layer and verifying that stripe patterns were steady during exposures. It was found that settle times depended heavily on the mirror step distance and longer settle times were needed after changing fringe orientation than after phase stepping.

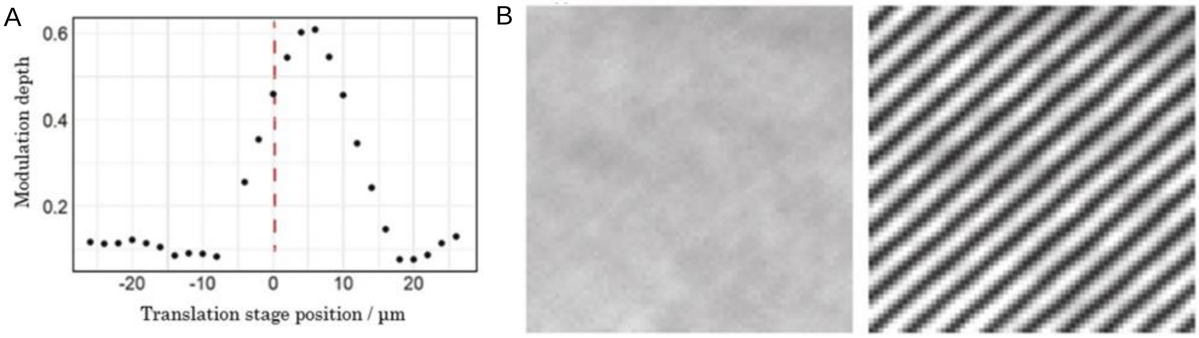

Supplementary Figure 2: Influence of path length on modulation depth. The effects of path length were measured using a low coherence length 647 nm diode laser. A: Plot of fringe contrast measured on a camera in an intermediate image plane as a function of translation stage position. Contrast was calculated as the peak to trough ratio for illumination patterns measured for a line profile along the pattern direction. B: Images acquired on the intermediate camera with un-optimized (left) and optimized (right) stage positions. Source data are provided as a Source Data file.

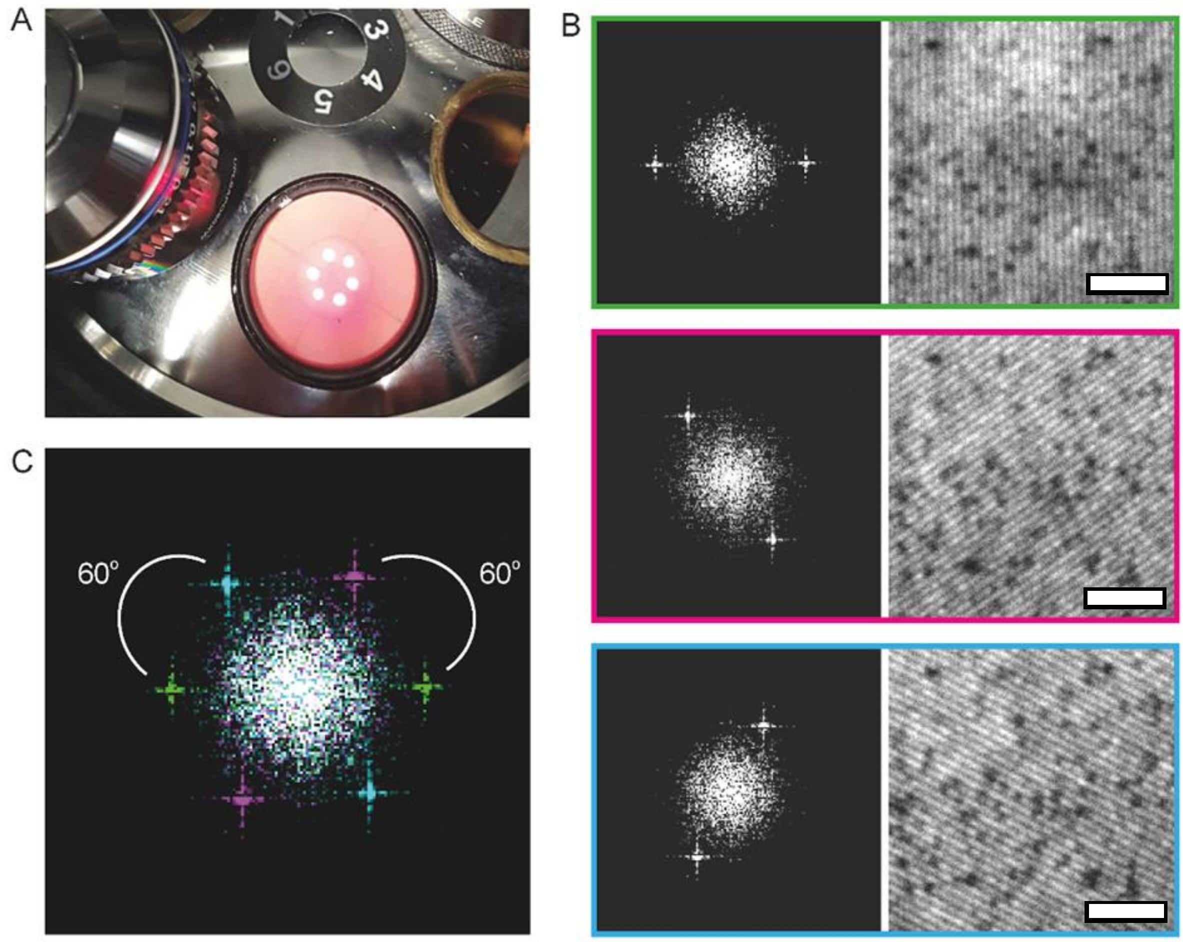

Supplementary Figure 3: Optimization of pattern orientation. The orientation of the fringe pattern is governed by the location of the beam foci at the back focal plane of the objective. A: An alignment target is used to provide an approximate estimate of mirror positions. B: The fringes are then viewed on a spin-coated fluorescent layer of 100 nm beads and the Fourier transform of the acquired image is then used to determine an appropriate fringe spacing. Scale bar is 5 µm C: The pattern rotation is optimized using the average Fourier transform for all pattern orientations.

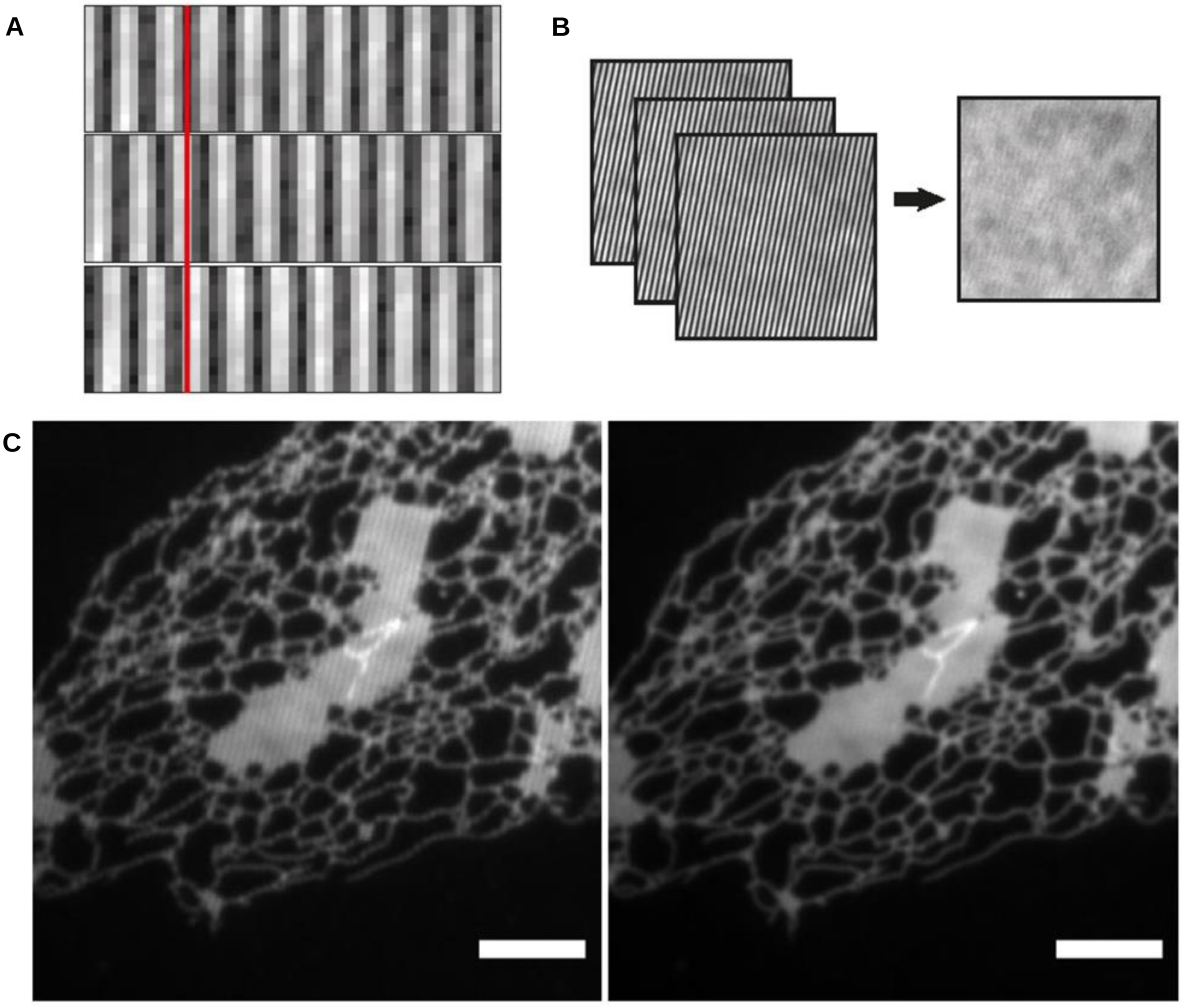

Supplementary Figure 4: Pattern phase optimization. A: Fringes formed by the 561 nm laser viewed on the intermediate camera. B: After correct selection of scan mirror voltages, the phases were checked by summing images acquired for all three pattern phase shifts to produce even and flat illumination. C: Widefield images of mApple labelled ER in live COS7 cells produced by summing images acquired at three pattern phases. Poorly (left) and well (right) adjusted phase voltages can be confirmed by checking for residual pattern modulation in the widefield image. Scale bar is 5 µm.

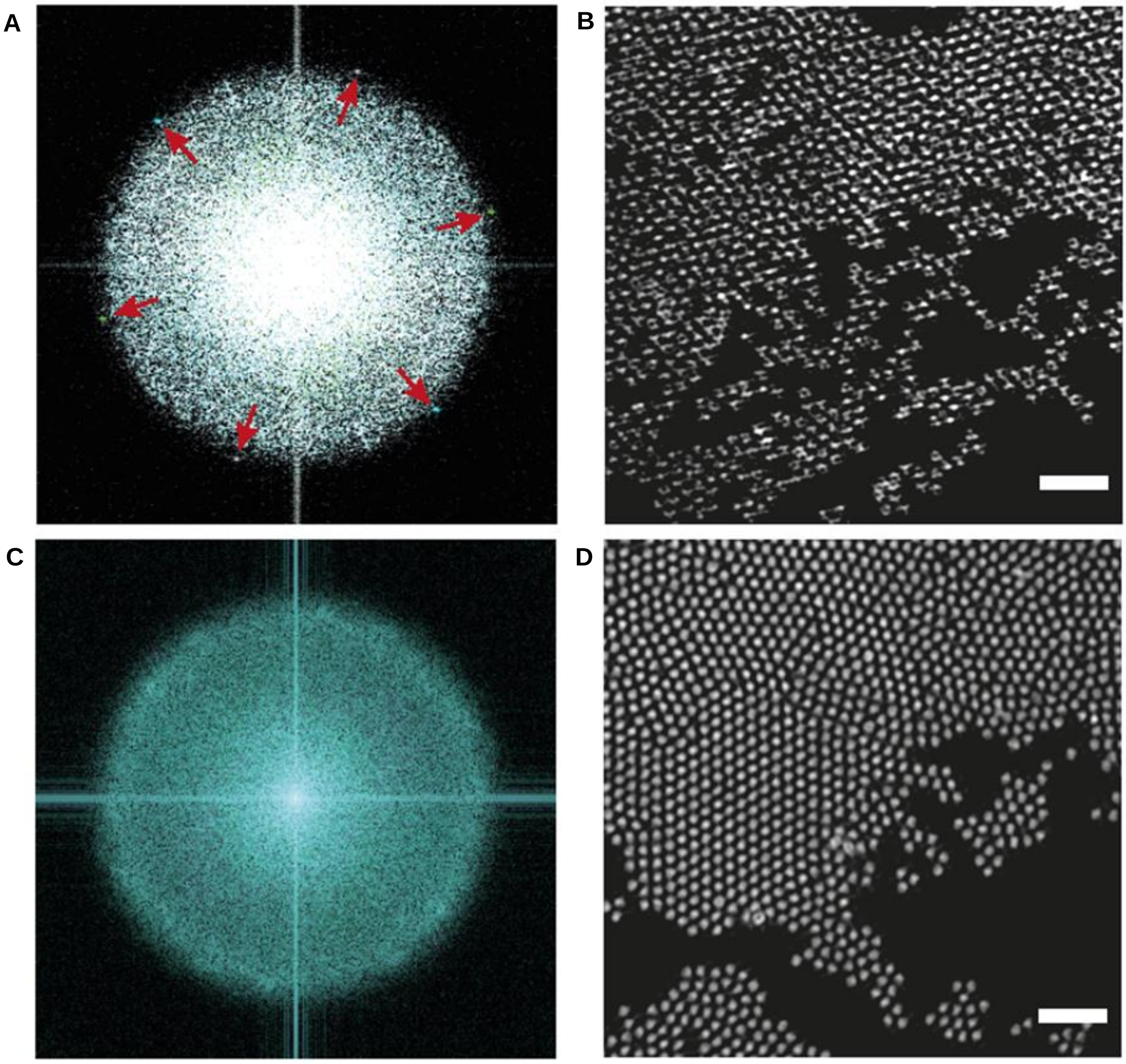

Supplementary Figure 5: Effects of phase shifting on reconstruction quality. A: Fourier spectrum of the widefield image calculated from the average of the 9 raw SIM frames. Peaks can be seen corresponding to stripes on the image. B: SIM reconstruction performed with FairSIM on SIM images with un-optimized phase steps. Uneven phase stepping results in significant reconstruction artifacts. C: Fourier spectrum of the widefield image calculated from the average of the 9 raw SIM frames. No residual peaks can be seen meaning a correct 2π/3 phase step has been achieved for all pattern orientations. D: SIM reconstruction performed with FairSIM on SIM images with optimized phase steps. Images of 200 nm beads were acquired upon excitation with the 561 nm laser line. Scale bar is 1 µm.

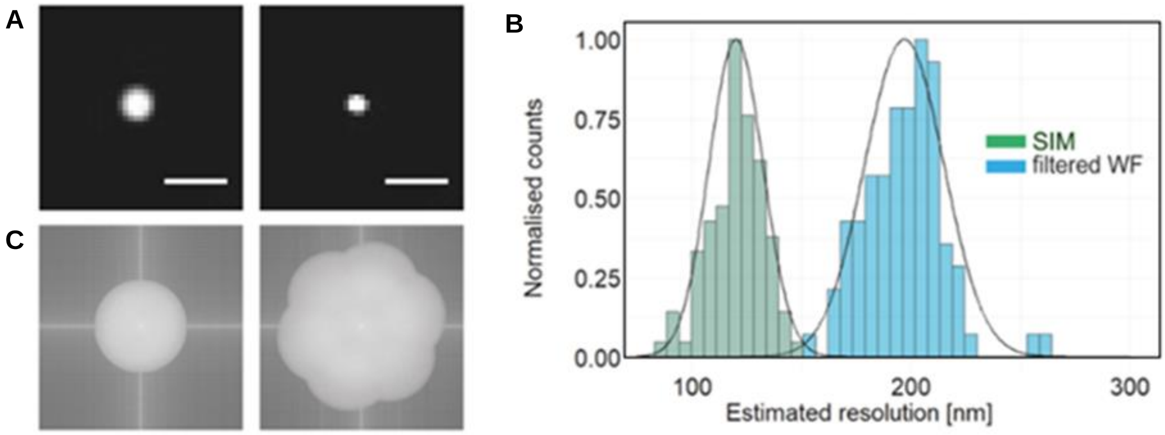

Supplementary Figure 6: Resolution estimation using 100 nm fluorescent microspheres. The resolution improvement provided by the instrument is quantified by measuring the width of sub-diffraction particles in reconstructed images. 100 nm fluorescent microspheres were excited by the 488 nm laser line super resolution reconstructions were performed in FairSIM. A: the particle size in MAI-SIM (right) is significantly reduced compared to the widefield image (left). Scale bars are 500 nm. B: Histogram of particle size measurements for 94 particles measured over 8 fields of view for N=1 bead preparation. Normal distribution fit to data suggests a mean diameter of 120 nm for particles imaged with SIM, while spheres in widefield mode have a diameter of 197 nm. C: Representative Fourier spectra for widefield image (left) and super-resolution reconstruction (right). The increased resolution is apparent as an extension of the frequency support region, visible as the central bright region. Source data are provided as a Source Data file.

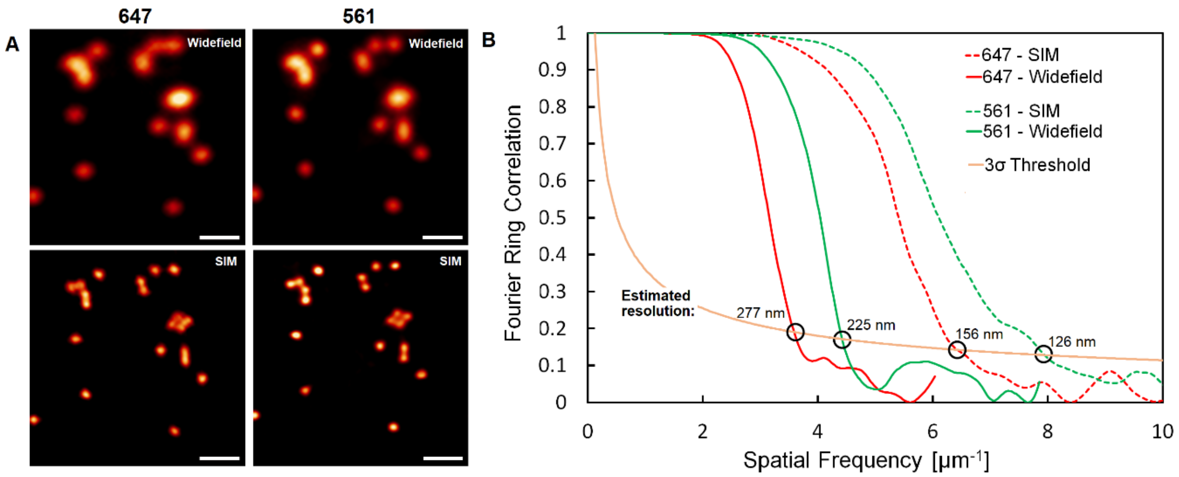

Supplementary Figure 7: Resolution estimation with Fourier ring correlation: A: Widefield and SIM images of 100 nm fluorescent microspheres imaged 647 nm and 561 nm excitation. SIM images were reconstructed using the inverse matrix approach. Scale bar is 500 nm. B: Fourier Ring Correlation (FRC) curves for the reconstructed microsphere images. The resolution is estimated from the intersection of the correlation curves (read and green) with the 3-sigma curve (orange).[5] The ca. 1.77 times resolution increase is in agreement with the resolution increase predicted from the fringe pattern spacing and pre-processing. FRC measurements were performed in imageJ using the available plugin.[5] Source data are provided as a Source Data file.

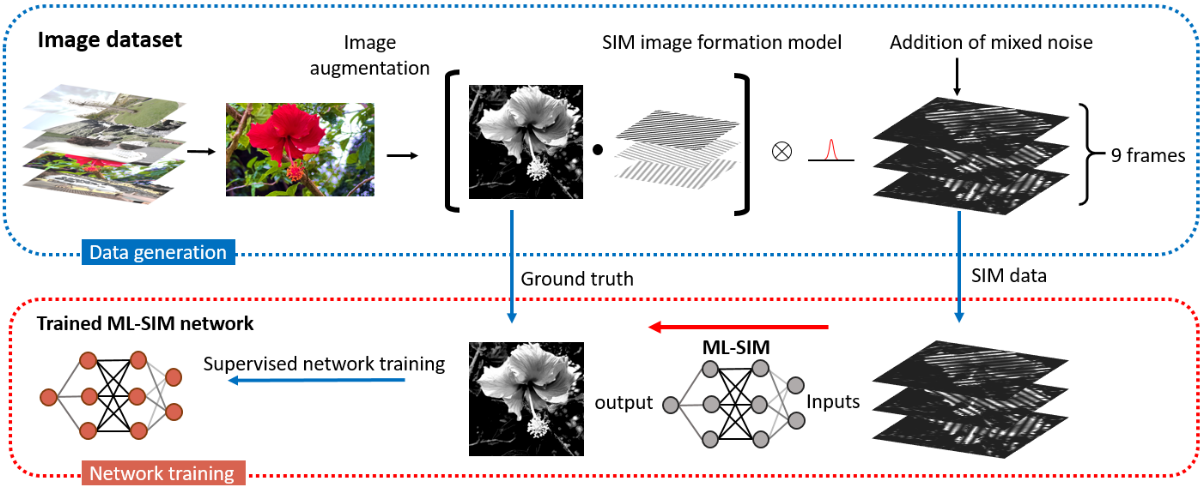

Supplementary Figure 8: Schematic of data generation and network training for ML-SIM. Training data is generated from the DIV2K image dataset [study [https://data.vision.ee.ethz.ch/cvl/DIV2K/].[6] Single images are taken randomly from the dataset undergo a greyscale conversion followed by resizing, rotation and thresholding to achieve a modal pixel value of 0. This has the effect of introducing high resolution features into the images as well as making them visually more similar to typical fluorescence microscopy images. Simulated SIM images are generated from the ground truth by multiplication with nine sinusoidal SIM fringes and convolution with an in-focus PSF. Crucially, and unlike previous ML-SIM implementations, these SIM fringes are generated with a high degree of phase shift error to simulate data acquired on the MAI-SIM system in simultaneous acquisition mode. An unmodulated background (not shown) can optionally be added to impart depth-sectioning capabilities to the model – akin to zero order suppression in classical reconstruction algorithms – although this step was omitted for the high phase error training data. A mixed Poisson-Gaussian noise model is then used to simulated photon shot noise and camera readout noise. Finally, salt and pepper noise is added to simulate hot pixels and dead pixels respectively. Details on the parameters used for generating the dataset can be found in the documentation in the project repository. This data generation pipeline is used to generate a training dataset consisting of 6000 simulated SIM images and the ML-SIM model is then trained by iterating through this dataset for 200 epochs. The advantages of this transfer learning approach are that very large datasets can be generated to ensure generalization and ideal ground truth images can be used to prevent the model from learning to replicate the imaging artefacts inevitably present in experimental datasets.

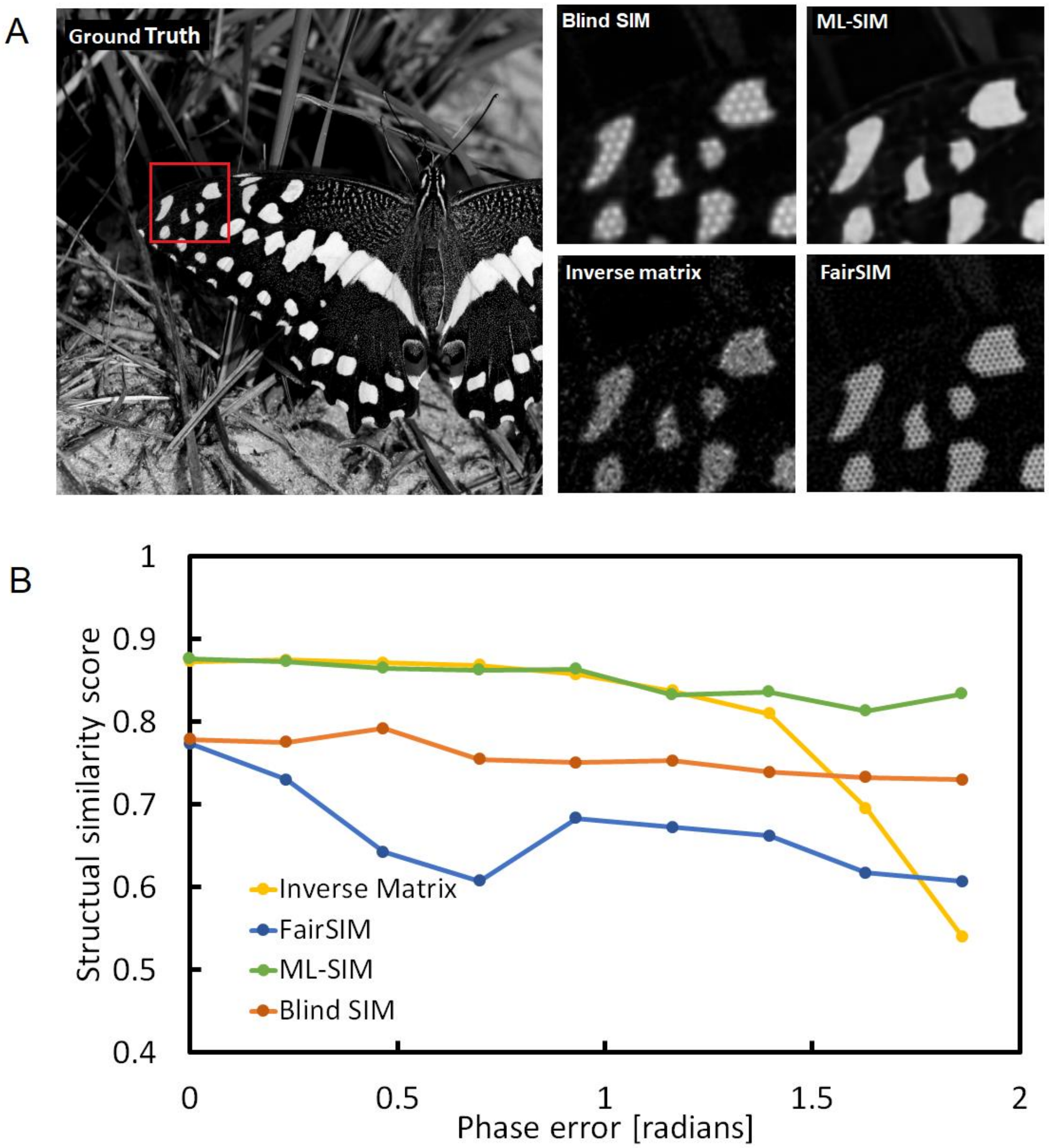

Supplementary Figure 9: Reconstruction performance with pattern phase error. A: Comparison of reconstruction techniques on simulated SIM image with an extreme phase error of 1.8 radians, equivalent to phase shifts of ~π/14. Image is taken from the validation subset of the DIV2K dataset.[7] Charles J. Sharp, CC BY-SA 4.0. B: Structural similarity (SSIM)[8] scores for the reconstructions measured against the ground truth image. SSIM scores were averaged over reconstructions of three simulated SIM datasets. SSIM measurements were performed in imageJ using a freely available plugin.[9] Source data are provided as a Source Data file.

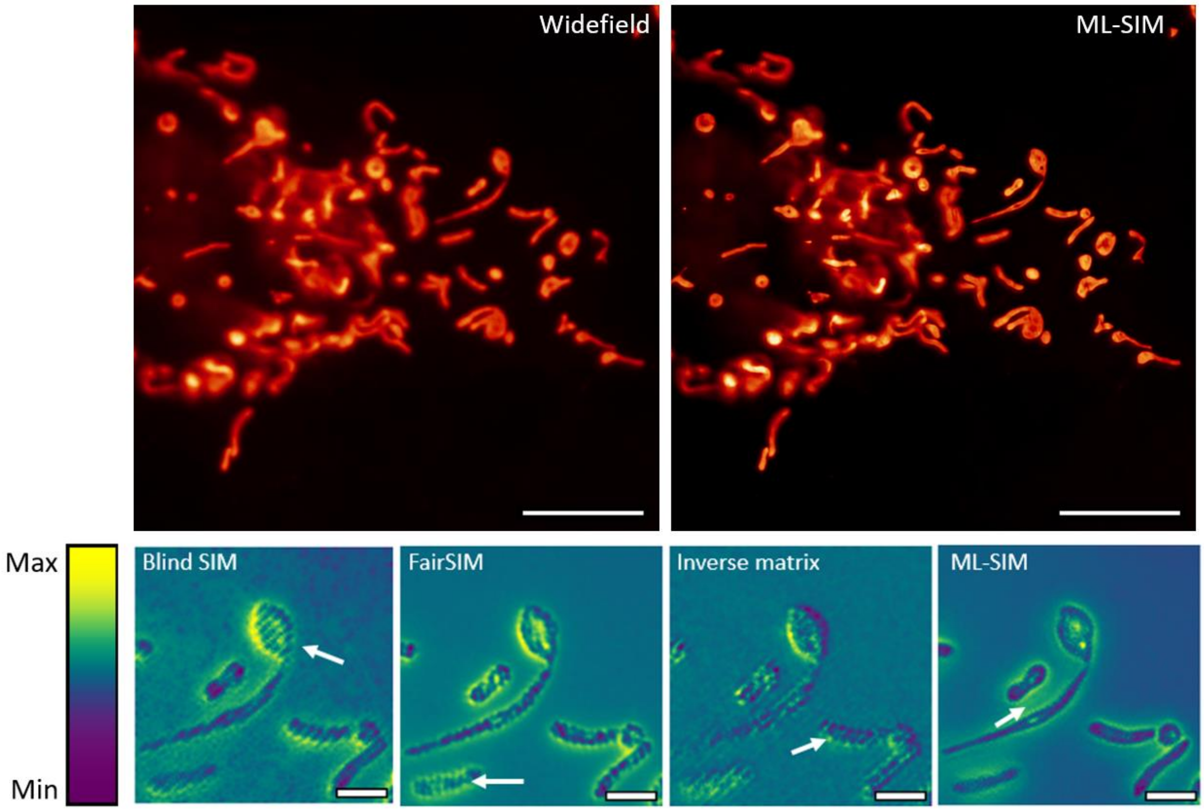

Supplementary Figure 10: Error map comparison of reconstruction quality. Top: Comparison of widefield and ML-SIM reconstructions of MAI-SIM data acquired of GFP labelled mitochondria in live COS7 cells. Pattern phase shifts were optimized for 561 nm excitation while imaging was performed with 488 nm excitation, leading to uneven phase stepping. Scale bar is 10 µm. Bottom: Normalized reconstruction error maps for Figure 4 calculated using NanoJ SQUIRREL. From left to right; Blind SIM: Diagonal striping artifacts (white arrow) are present in the image. As Blind SIM does not require specific illumination patterns, these artifacts can be attributed to an improper initial estimate of the sample, indirectly caused by irregular phase shifts. FairSIM: Honeycombing and vertical stripe artifacts are present (white arrow) which result from irregular phase shifts, poor estimates of pattern frequency, and reduced modulation depth. Inverse matrix: As with Blind SIM, the principal artifact is diagonal striping (white arrow). In the case of the inverse matrix reconstruction, this is a result of pattern phase shifting being irregular and beyond what the algorithm is capable of handling. ML-SIM: The error in ML-SIM reconstructions is primarily limited to the background (white arrow) where out of focus signal dominates and not the key sample structures. Scale bar is 2 µm.

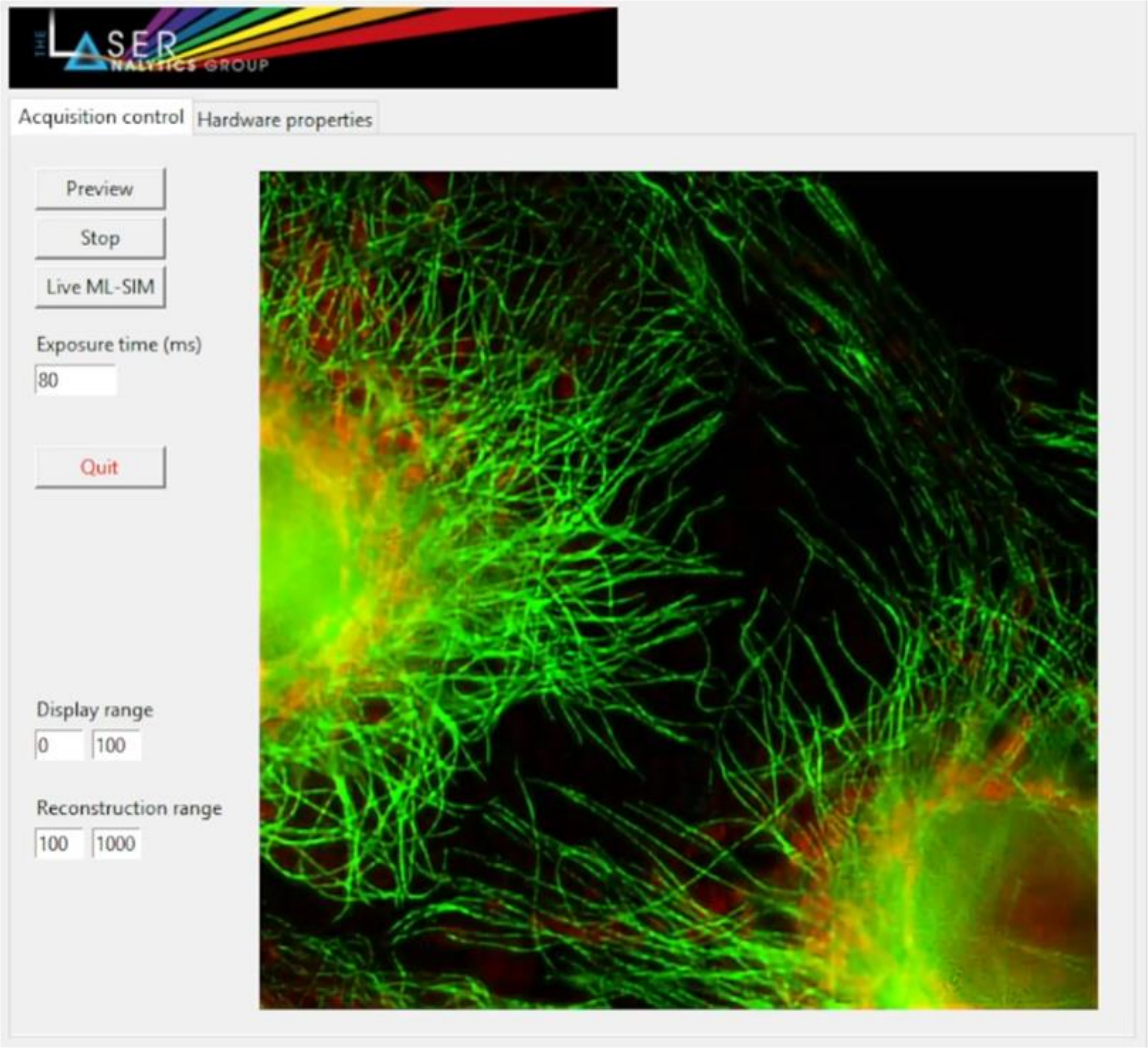

Supplementary Figure 11: GUI for live ML-SIM reconstruction. Image shows a 2-color reconstruction of fixed COS7 cells over a 44 µm × 44 µm FOV. Immunostained AlexaFluor647-Tubulin and GPF-tagged mitochondria are shown in magenta and cyan, respectively. Video of operation can be found in the figshare repository [https://figshare.com/projects/MAI-SIM/140008].

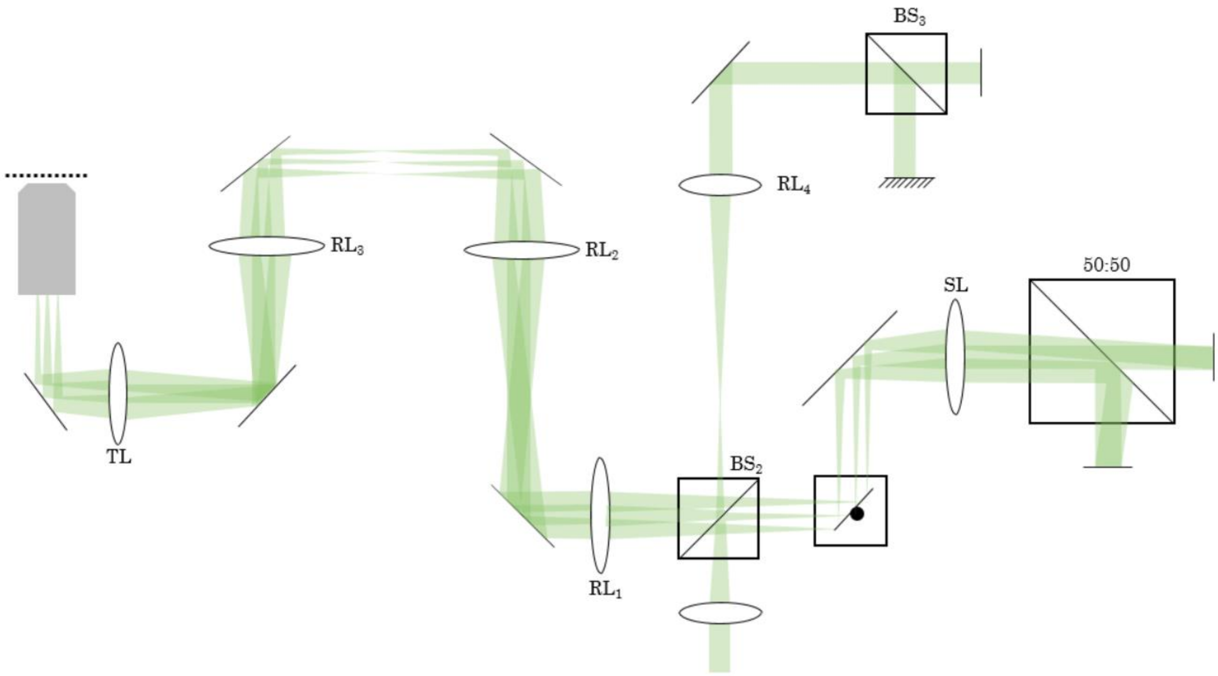

Supplementary Figure 12: Proposed optical setup for 3D MAI-SIM. By replacing the D-mirror with a second beam splitter cube (BS2), an additional arm can be introduced to the interferometer to allow for 3-beam interference in the sample. A third beam splitter (BS3) preserves the path length of the beams and ensures equal beam intensity.

Supplementary Note 2 - MAI-SIM alignment and calibration

The Michelson interferometer: The Michelson interferometer is a commonly used amplitude splitting interferometer which, due to its simplicity and high accuracy, is popular in a wide range of applications.[1] In such a system, a beam splitter divides a primary wave into two beams which are subsequently reflected by two mirrors in the corresponding optical path. The two returning beams interfere after being recombined by a condensing lens and create periodic intensity fringes, when incident on a screen or detector. A tilted mirror pair leads to the formation of so-called wedge fringes. The angle of mirror inclination determines the frequency of the pattern, while the path length difference (among other factors) defines the pattern contrast for non-monochromatic sources. For a clean sinusoidal fringe pattern with a high contrast, good quality light sources with high thermal stability and a narrow bandwidth are essential. Temporal coherence of the two interfering beams is important for the pattern quality as high contrast interference patterns will only be visible if the optical path length difference (i.e. twice the wedge thickness) between the interfering beams is smaller than the coherence length of the light source. The alignment of the interferometer mirrors is greatly facilitated when laser coherence lengths of several mm or more are used. To adjust the path length differences and optimize the fringe contrast in the MAI-SIM system, a camera was mounted in an intermediate image plane to directly observe changes to the illumination pattern. The optical path length difference was then adjusted for each chosen set of mirrors, using the micrometer translation stages on which one of the mirrors was mounted.

Pattern alignment: Pattern orientation and period are controlled by directing the beams into the appropriate positions at the back of the objective by adjusting the three pairs of small mirrors. For precise alignment, a fluorescent bead monolayer was viewed and the stripes brought into focus (Supplementary Figure 3). A live FFT implemented through MATLAB was then used to view the frequency components of the stripes in real time, permitting precise alignment of the spots in the back focal plane of the objective.

Pattern orientation change and phase stepping: The system utilizes one scan mirror to achieve both rotation of the fringe pattern in the sample (by stepping from one mirror pair to the next) and phase stepping (by small angular displacements). To determine the step size required for adequate phase shifts, a camera is placed in the intermediate image plane, once the spot position and pattern frequency have been optimized. This enables a direct observation of the pattern and voltages supplied to the scan mirror are adjusted until a phase shift of approximately 2π/3 is observed on the camera. After this coarse visual adjustment, a finer adjustment is achieved by summing the images from the three phase steps and confirming a cancellation of the stripes in the resulting image, which should be uniform in intensity (Supplementary Figure 5). If sequential imaging of different color channels is to be performed, this process can be optimized for each color separately. For simultaneous multicolor imaging in conjunction with the image splitter, the central wavelength (in our case 561 nm) is chosen to optimize the phase steps. While this enables fast imaging, the phase steps will no longer be 2π/3 for the other colors, and inverse or Fourier based reconstruction methods may no longer work, leading to reconstruction

artifacts (Supplementary Figure 6). As an additional control correct pattern shifting, the process described above is repeated by imaging the pattern shifts in the actual sample plane.

Detection optics and channel registration: For a coarse alignment of the detection system and channel registration, a ruled grid slide (Thorlabs, R1L3S2P) was illuminated with the brightfield lamp of the system, brought into focus, and centered on the camera. The filter cubes of the image splitter, containing 2 dichroic mirrors and 3 emission filters, were inserted and the adjustable mirrors in the device were aligned such that the same region of the slide was visible in all three color channels. Fine adjustment of the channels was performed before each measurement. The required dichroic mirrors and emission filters were inserted into the filter cubes according to the fluorophores in the sample. 100 nm diameter fluorescent beads which emit in all three color channels were imaged onto the camera and the mirrors in the image splitting devices were adjusted for a chosen cluster of beads to line up in the same position in all channels. Images were then recorded in two or three colors simultaneously and fine registration was performed after acquisition using a calibration image of a sparse 100 nm bead sample.

Supplementary Note 3 - Reconstruction methods

During high-speed multicolor imaging, low signal levels and imperfect phase stepping make reconstruction challenging with conventional techniques. We compare four reconstruction methods on simulated and experimental MAI-SIM data. FairSIM[10] and the inverse matrix[11] methods represent implementations of the original SIM reconstruction algorithm proposed by Gustafsson.[12] In both cases the pattern periods and orientations are determined by iterative cross correlation of the Fourier transforms of the data and the two techniques differ by the phase estimation approaches used. By assuming an equidistant phase separation (i.e., phases separated by 2π/3), FairSIM reduces the parameter estimation process to extraction of only a single global phase offset. In contrast, the inverse matrix approach recovers the phases by analytically solving a trigonometric linear equation without the need for iterative optimization, recovering absolute phase values for each image. Frequency reassignment is performed with the same method for both FairSIM and the inverse matrix approach and the methods differ only in the phases used for this reassignment. The Blind SIM approach used was a MATLAB implementation of a joint reconstruction strategy where both the reconstructed image and the illumination pattern are iteratively optimized from initial estimates.[13] The initial estimate for the sample was taken as the average of the nine raw SIM frames and an initial estimate of the pattern was calculated by element-wise division of the sample estimate by each raw frame.

The machine learning approach used to address uneven phase stepping is based on previously described work using transfer learning to train a convolutional neural network (CNN) on simulated SIM images.[14] Training data were generated by modeling the SIM image formation process on artificial "samples" taken as 512 × 512 pixel patches from images in the high-resolution DIV2K image dataset.[6] The imaging parameters for pixel size; excitation and emission wavelengths; fringe spacing, orientation and modulation depth; and imaging depth were randomized within the bounds of the expected imaging conditions. Random Poisson and Gaussian noise were then added to the images. The model was then trained in a supervised fashion where the original image patches were used as the ground-truth. The Adam optimizer and mean-square error were used for training and the model was trained on 5000+ samples for 300 epochs. Python code and the PyTorch[15] machine learning library were used throughout. The code used to generate the training data, train the ML-SIM models, and evaluate the model on experimental data is available in the GitHub repository.[16] Three models with different network architectures are available for evaluation in this repository. The first is a broad model with 96 feature maps, 10 residual blocks, and 3 residual groups.[14] The second is a narrower model which can be evaluated faster consisting of 48 feature maps, 10 residual blocks, and 3 residual groups. Live ML-SIM reconstructions were performed with the shallower model for best execution speed. Static ML-SIM reconstructions were performed using the broader model and reconstructions of dynamic structures were performed after acquisition using an experimental visual transformer model which has improved performance on dynamic structures at the expense of increased reconstruction times.[17]

To evaluate the performance of the techniques on data with uneven phases steps we first quantitatively measure the Resolution Scaled Pearson (RSP) coefficient and the Resolution Scaled Error (RSE) implemented through the NanoJ plugin for imageJ.[18] These provide a measure of the likelihood that a reconstruction could represent the real sample based on comparison between a predicted and real diffraction limited image. RSP values range from 0 to 1, with 1 representing a perfect correlation and ideal reconstruction. The RSE is a measure of the mean square error values between the predicted widefield image and the observed widefield image, with a lower value showing a better reconstruction. Table S1 compares the performance of the reconstruction techniques on simulated SIM data with ideal and imperfect pattern phase stepping. The use of simulated targets allows for the effects of only phase errors to be assessed without other factors (such as background fluorescence and artifacts in the reference image) affecting the reconstruction quality metric. The code to generate the simulated images can be found on the project GitHub[16] and the images used can be found via the figshare repository [https://figshare.com/projects/MAI-SIM/140008]. In all cases the reconstructions have performed better on data with the correct phase steps, which is expected. Notably, ML-SIM out-performs all methods except for the inverse matrix method when ideal phase shifts are used. For this reason, the inverse matrix method was used for sequential data and ML-SIM reconstruction was used on simultaneous acquisitions.

Table S1: Reconstruction quality measured by RSP and RSE. For all reconstruction techniques, reconstructions are better on data with correct phase stepping. In the case of FairSIM this is expected as phases cannot be calculated. For Blind SIM, this discrepancy can be attributed to the initial estimate of the sample containing residual fringe patterns. For the inverse matrix and ML-SIM methods, reduced performance is likely a result of reduced information being available on regions of the sample unevenly illuminated or residual striping in the widefield image. Source data are provided as a Source Data file.

Method	Bad phases		Good phases	
	RSP	RSE	RSP	RSE
ML-SIM	0.991	1497	0.994	1134
Blind SIM	0.978	2280	0.983	1960
FairSIM	0.975	2419	0.992	1362
Inverse Matrix	0.980	2196	0.995	1085

References:

1. P. Hariharan. *Basics of Interferometry*. (Elsevier, 2007). doi:10.1016/B978-0-12-373589-8.X5000-7.
2. O'Holleran, K. & Shaw, M. Polarization effects on contrast in structured illumination microscopy. *Opt Lett* **37**, 4603 (2012).
3. Ejlli, A., Della Valle, F. & Zavattini, G. Polarisation dynamics of a birefringent Fabry–Perot cavity. *Applied Physics B* **124**, 22 (2018).
4. Edelstein, A. D. *et al.* Advanced methods of microscope control using μManager software. *J Biol Methods* **1**, e10 (2014).
5. Nieuwenhuizen, R. P. J. *et al.* Measuring image resolution in optical nanoscopy. *Nat Methods* **10**, 557–562 (2013).
6. Agustsson, E. & Timofte, R. NTIRE 2017 Challenge on Single Image Super-Resolution: Dataset and Study. in *2017 IEEE Conference on Computer Vision and Pattern Recognition Workshops (CVPRW)* 1122–1131 (IEEE, 2017). doi:10.1109/CVPRW.2017.150.
7. Lambert, T. J. & Waters, J. C. Navigating challenges in the application of superresolution microscopy. *Journal of Cell Biology* **216**, (2017).
8. Wang, Z., Bovik, A. C., Sheikh, H. R. & Simoncelli, E. P. Image Quality Assessment: From Error Visibility to Structural Similarity. *IEEE Transactions on Image Processing* **13**, 600–612 (2004).
9. Prieto, G., Chevalier, M. & Guibelalde, E. SSIM Index as a Java plugin for ImageJ. Department of Radiology, Faculty of Medicine. Universidad Complutense. Madrid.
10. Müller, M., Mönkemöller, V., Hennig, S., Hübner, W. & Huser, T. Open-source image reconstruction of super-resolution structured illumination microscopy data in ImageJ. *Nat Commun* **7**, 10980 (2016).
11. Cao, R. *et al.* Inverse matrix based phase estimation algorithm for structured illumination microscopy. *Biomed Opt Express* **9**, 5037 (2018).
12. Gustafsson, M. G. Surpassing the lateral resolution limit by a factor of two using structured illumination microscopy. *J Microsc* **198**, 82–87 (2000).
13. Labouesse, S. *et al.* Joint Reconstruction Strategy for Structured Illumination Microscopy With Unknown Illuminations. *IEEE Transactions on Image Processing* **26**, 2480–2493 (2017).
14. Christensen, C. N., Ward, E. N., Lu, M., Lio, P. & Kaminski, C. F. ML-SIM: universal reconstruction of structured illumination microscopy images using transfer learning. *Biomed Opt Express* **12**, 2720 (2021).
15. Paszke, A. *et al.* PyTorch: An Imperative Style, High-Performance Deep Learning Library. in *Advances in Neural Information Processing Systems 32* (eds. Wallach, H. et al.) 8024–8035 (Curran Associates, Inc., 2019).
16. Ward, E. N. *et al.* MAI-SIM: interferometric multicolor structured illumination microscopy for everybody. *Zenodo* Preprint at https://doi.org/10.5281/zenodo.7123696 (2022).
17. Christensen, C. N., Lu, M., Ward, E. N., Lio, P. & Kaminski, C. F. Spatio-temporal Vision Transformer for Super-resolution Microscopy. (2022).
18. Culley, S. *et al.* NanoJ-SQUIRREL: quantitative mapping and minimization of super-resolution optical imaging artifacts. *Nat Methods* **15**, 263–266 (2018).
