# Supplementary material for: Machine learning assisted interferometric structured illumination microscopy for dynamic biological imaging
Source: Nat Commun. 2022 Dec 21;13:7836. doi: 10.1038/s41467-022-35307-0 (PMC9772218; doi:10.1038/s41467-022-35307-0)
Supplement: Supplementary file 3 — Reporting Summary [file 41467_2022_35307_MOESM3_ESM.pdf]

nature portfolio

Corresponding author(s): Kaminski, CF

Last updated by author(s): 2022/11/05

Reporting Summary

Nature Portfolio wishes to improve the reproducibility of the work that we publish. This form provides structure for consistency and transparency in reporting. For further information on Nature Portfolio policies, see our Editorial Policies and the Editorial Policy Checklist.

Statistics

For all statistical analyses, confirm that the following items are present in the figure legend, table legend, main text, or Methods section.

n/a	Confirmed	
☐	☒	The exact sample size (n) for each experimental group/condition, given as a discrete number and unit of measurement
☐	☒	A statement on whether measurements were taken from distinct samples or whether the same sample was measured repeatedly
☒	☐	The statistical test(s) used AND whether they are one- or two-sided<br>*Only common tests should be described solely by name; describe more complex techniques in the Methods section.*
☒	☐	A description of all covariates tested
☒	☐	A description of any assumptions or corrections, such as tests of normality and adjustment for multiple comparisons
☒	☐	A full description of the statistical parameters including central tendency (e.g. means) or other basic estimates (e.g. regression coefficient) AND variation (e.g. standard deviation) or associated estimates of uncertainty (e.g. confidence intervals)
☒	☐	For null hypothesis testing, the test statistic (e.g. F, t, r) with confidence intervals, effect sizes, degrees of freedom and P value noted<br>*Give P values as exact values whenever suitable.*
☒	☐	For Bayesian analysis, information on the choice of priors and Markov chain Monte Carlo settings
☒	☐	For hierarchical and complex designs, identification of the appropriate level for tests and full reporting of outcomes
☒	☐	Estimates of effect sizes (e.g. Cohen's d, Pearson's r), indicating how they were calculated

Our web collection on statistics for biologists contains articles on many of the points above.

Software and code

Policy information about availability of computer code

Data collection	Image data were collected from the camera using micromanager (version 2.0.1). Camera triggering and laser control were achieved though a custom made interface built using LabVIEW (version 21.0.1). Post-processing was performed using the open-source software ImageJ (version 2.35), NanoJ-SQUIRREL (version 2.1RC1) and FairSIM (original release).
Data analysis	Data analysis was completed using custom software which is referenced in the text. The GitHub repository can be found at (https://github.com/edward-n-ward/MAI-SIM) and the static release is made available through Zenodo at (https://doi.org/10.5281/zenodo.7123696). MATLAB programs were built and run using MATLAB 2021b. Python version 3.9 was used for the live view implementation. Machine learning training and inference were performed in Python 3.9 using the PyTorch library (version 1.10.1_cuda11.3)

For manuscripts utilizing custom algorithms or software that are central to the research but not yet described in published literature, software must be made available to editors and reviewers. We strongly encourage code deposition in a community repository (e.g. GitHub). See the Nature Portfolio guidelines for submitting code & software for further information.

Data

Policy information about availability of data

All manuscripts must include a data availability statement. This statement should provide the following information, where applicable:

- Accession codes, unique identifiers, or web links for publicly available datasets
- A description of any restrictions on data availability
- For clinical datasets or third party data, please ensure that the statement adheres to our policy

The image data that support the findings of this study are available in the Figshare repository https://figshare.com/projects/MAI-SIM/140008
The DIV2K dataset (https://data.vision.ee.ethz.ch/cvl/DIV2K/) was used for network training.

Human research participants

Policy information about studies involving human research participants and Sex and Gender in Research.

Reporting on sex and gender	N/A
Population characteristics	N/A
Recruitment	N/A
Ethics oversight	N/A

Note that full information on the approval of the study protocol must also be provided in the manuscript.

Field-specific reporting

Please select the one below that is the best fit for your research. If you are not sure, read the appropriate sections before making your selection.

☒ Life sciences ☐ Behavioural & social sciences ☐ Ecological, evolutionary & environmental sciences

For a reference copy of the document with all sections, see nature.com/documents/nr-reporting-summary-flat.pdf

Life sciences study design

All studies must disclose on these points even when the disclosure is negative.

Sample size	A total of seven biological samples were used for the data presented. Repeats were taken by imaging different regions of the same sample. The number of repeats acquired for each sample (N) is indicated in the figure descriptions.
Data exclusions	Image data were not used for live cell imaging where transfection was unsuccessful.
Replication	For each sample, repeats were taken by imaging different regions. The number of repeats acquired for each sample (N) is indicated in the figure descriptions. All attempts at replication were successful.
Randomization	No randomisation was necessary as samples were not compared to each other.
Blinding	No blinding was necessary as samples were not compared to each other.

Reporting for specific materials, systems and methods

We require information from authors about some types of materials, experimental systems and methods used in many studies. Here, indicate whether each material, system or method listed is relevant to your study. If you are not sure if a list item applies to your research, read the appropriate section before selecting a response.

Materials & experimental systems

n/a	Involved in the study	
☐	☒	Antibodies
☐	☒	Eukaryotic cell lines
☒	☐	Palaeontology and archaeology
☒	☐	Animals and other organisms
☒	☐	Clinical data
☒	☐	Dual use research of concern

Methods

n/a	Involved in the study	
☒	☐	ChIP-seq
☒	☐	Flow cytometry
☒	☐	MRI-based neuroimaging

Antibodies

Antibodies used	mouse anti-beta-tubulin: abcam, ab131205, clone number [1E1-E8-H4], lot number GR3247797-1<br>rabbit anti-calnexin: abcam, ab22595, polyclonal, lot number GR3359380-1<br>goat anti-mouse conjugated to AlexaFluor568, invitrogen, A21124, clone number [12H6], lot number: 2300937<br>goat anti-mouse conjugated to AlexaFluor647, invitrogen, A21235, polyclonal, lot number: 784584<br>goat anti-rabbit conjugated to AlexaFluor568, invitrogen, A11036, polyclonal, lot number: 2155282
Validation	Mouse anti-beta-tubulin validated by manufacturer for COS7 cells with Western Blot. Rabbit anti-calnexin demonstrated for african green monkey in "Allen S.J. et al. PLOS one 9(1)".

Eukaryotic cell lines

Policy information about cell lines and Sex and Gender in Research

Cell line source(s)	COS-7 cells (male) were obtained from commercial supplier (American Type Culture Collection, ATCC CRL-1651).
Authentication	No authentication was performed as cell lines were obtained from commercial supplier.
Mycoplasma contamination	Cells were tested and found negative for Mycoplasma. Mycoplasma test was performed using MycoAlert® Mycoplasma Detection Kit from Lonza Ref: LT07-418
Commonly misidentified lines (See ICLAC register)	None
